# Computed Tomography Imaging in ILD: New Trends for the Clinician

**DOI:** 10.3390/jcm11195952

**Published:** 2022-10-09

**Authors:** Gregor S. Zimmermann

**Affiliations:** 1 Department of Respiratory Medicine, InnKlinikum, Academic Hospital of the Technical University of Munich, 84453 Muehldorf am Inn, Germany; gregor.zimmermann@tum.de; 2 Department of Internal Medicine I, School of Medicine, University Hospital Rechts der Isar, Technical University of Munich, 81675 Munich, Germany

Today, radiological methods are an integral part of diagnostics in lung diseases, and they provide important information regarding the evaluation of interstitial lung diseases (ILDs) [1]. Using this information, ILDs can be classified more precisely, considering a detailed workup and the exclusion of alternate diagnoses [1]. In addition to established imaging methods that are being continuously re-evaluated under new aspects in recent years, entirely new imaging methods are expected to become available in the next few years.

Imaging in interstitial lung diseases is mainly restricted to high-resolution thoracic computed tomography scans as the established gold standard. In addition to the morphological information from different patterns in CT scans, new aspects such as quantitative information can also be acquired via CT and may be integrated into the clinical routine [2].

Quantitative imaging data offers potential improvements in diagnostic accuracy and mortality prediction [3,4,5,6,7]. There are several methods for quantification. For many years, semi-quantitative methods were used by different investigators using a standardized visual scoring system. However, higher reproducibility may be achieved using automated methods, which demonstrate a stronger correlation of prognoses than traditional visual scores [3,8,9].

Automated or semiautomated quantitative imaging may reduce interobserver variability by providing reproducible measurements and may be used in comparative studies or longitudinal evaluations [3,10,11]. While longitudinal evaluations have provided promising results, the diagnostic significance using quantitative measurements to characterize the particular ILD at the first time of diagnosis remains unclear [3,4,11,12]. The lack of standardization and individual technical confounding factors due to artifacts in image acquisition challenges the diagnostic evaluation of ILD patients with these tools.

A frequently used method for automatic quantification is Automated Computer-Aided Lung Informatics for Pathology Evaluation and Rating (CALIPER) volumetric analysis [11,12,13]. CALIPER analysis reveals detailed information about the structural composition of the lungs in ILD patients [11,13]. By using volumetric recordings of individual parenchymal patterns such as reticulations, ground-glass opacities, honeycombing, low attenuation area, or normal tissue, these data can be acquired for each lung [14]. An automated segmentation of pulmonary-vascular-related structures in the lung parenchyma calculated using CALIPER may provide valuable information regarding differentiation and mortality in ILDs [12,14].

Due to its standardized evaluation, high reproducibility, and prognostic significance, computer-based quantitative evaluation in ILDs is suitable for outcome assessment and is suitable also as a potential endpoint in clinical trials [4,11].

However, despite its potential to provide information regarding the progression and prognosis of ILDs, quantitative imaging technology is rarely used in the clinical routine, and its use is mostly limited to scientific studies and trials. So far, clinical use is very rare despite its many potential applications.

In recent years, a new low-dose imaging method in radiological imaging has been developed, which can investigate structural changes in the lung parenchyma in the context of a dark-field chest X-ray scan [15,16,17]. Dark-field X-ray imaging exploits the wave properties of X-rays for contrast formation by visualizing small-angle scattering occurring at air–tissue interfaces, e.g., in pulmonary alveoli [18]. High dark-field signals correlate with the number of these interfaces, e.g., alveolar walls. In these types of studies, improved visualization could be achieved compared to that obtained using conventional chest X-ray (CXR). Given the fact that conventional chest X-ray is not suitable in the early recognition or surveillance of structural lung changes, this new technique may provide a new potential in imaging the lung [19]. There have been studies in humans with emphysema showing a better correlation to the DLCO with dark-field chest X-ray than CT-based scores [18,20]. This technique enables more information to be acquired regarding the ultrastructure of the lung than through conventional chest X-ray, with only marginally more elevation exposure [18]. Hence, dark-field imaging may be a potential tool for the further quantification of lung structure changes in the future [18,21,22,23].

In patients with various lung pathologies, by utilizing dark-field chest X-ray, these structural changes could be shown more clearly compared to conventional CXR. Compared to a CT scan, the use of dark-field chest X-ray exhibited a signal loss in the most affected areas in a patient with lymphangioleiomyomatosis, a rare disease presenting cystic changes in the lung parenchyma [23]. In a patient with combined pulmonary fibrosis and emphysema, a modulation in the dark-field chest X-ray signal according to the structural changes and their location could be shown [22]. Due to the complexity of the construction, for a long time, this technology was only feasible in the same setup as a conventional CXR and only provided two-dimensional images. The use of this novel technique is still limited to prototypes [16,17,18,20]. The limitation of the three-dimensional resolution could be overcome by the recent development of dark-field technology integration into a computed tomography scanner. It was shown that this integration significantly increased the information content in the context of parenchymal changes in the lung, as shown in small animal studies [24]. Recently, a prototype was developed that enables dark-field computed tomography scans on human-sized anthropomorphic body phantoms [25].

In the field of radiological imaging, a variety of methods have been developed in recent years. In addition to the abovementioned promising methods, much progress has been made in many areas of imaging, including MRI imaging and nuclear medicine, which offer considerable improvement in the provision of information regarding the lungs.

There is a need to integrate these new methods and their automated quantification into clinical practice. New diagnostic methods, such as dark-field imaging, may provide supplementary information in the assessment of imaging in patients with ILDs in the future.

The consistent development of thoracic imaging and its integration into everyday clinical practice is crucial, and an improvement in the care of patients with ILDs may be achieved.

## Abbreviations

CALIPER	Computer-Aided Lung Informatics for Pathology Evaluation and Rating
CT	computed tomography
CXR	chest X-ray
DLCO	diffusion capacity of the lung for carbon monoxide uptake
ILD	interstitial lung disease
